# Patterns of Social Connection Among Older Adults in England

**DOI:** 10.1001/jamanetworkopen.2024.51580

**Published:** 2024-12-23

**Authors:** Feifei Bu, Daisy Fancourt

**Affiliations:** 1 Department of Behavioural Science and Health, Institute of Epidemiology and Health Care, University College London, London, United Kingdom

## Abstract

**Importance:**

Issues related to social connection are increasingly recognized as a global public health priority. However, there is a lack of a holistic understanding of social connection and its health impacts given that most empirical research focuses on a single or few individual concepts of social connection.

**Objective:**

To explore patterns of social connection and their associations with health and well-being outcomes.

**Design, Setting, and Participants:**

This cohort study included participants aged 50 years and older from the fourth wave of the English Longitudinal Study of Aging (2008-2009). Machine learning cluster analysis and regression analysis were used. The analyses were performed from January to July 2024.

**Exposure:**

Social connection clusters informed by the cluster analysis.

**Main Outcomes and Measures:**

This study considered outcomes related to mental health (depression), hedonic (life satisfaction, pleasure) and eudaimonic (self-realization) well-being, general health (self-reported health), and health behavior (moderate or vigorous physical activity). Key confounders, identified using directed acyclic graphs, including age, sex, ethnicity, education, social class, and wealth, were controlled for.

**Results:**

Among 7706 participants aged 50 years and older (mean [SD] age, 64.7 [9.6] years; 4248 [55.1%] female; 7536 [97.8%] White), 5 clusters were identified, including disconnected (974 [12.6%]), gapped structure/poor function (1109 [14.4%]), gapped structure/high function (1582 [20.5%]), poor function/mixed quality (1501 [19.5%]), and highly connected (2540 [33.0%]). All clusters had poorer outcomes compared with the highly connected cluster (eg, depression among individuals in disconnected vs highly connected clusters: odds ratio [OR], 2.73; 95% CI, 2.24 to 3.33), many of which persisted after controlling for baseline outcome (eg, depression among individuals in disconnected vs highly connected clusters: OR, 1.95; 95% CI, 1.57 to 2.43). The difference was smallest between the highly connected and gapped structure/high function clusters across most outcomes (eg, depression among individuals in gapped structure/high function vs highly connected: OR, 1.34; 95% CI, 1.10-1.64; after controlling for baseline outcome: OR, 1.28; 95% CI, 1.03-1.59).

**Conclusions and Relevance:**

This cohort study highlights the importance of considering multidimensional measures of social connection and understanding the nuance of its heterogenous patterns. Understanding the typologies of social connection has substantial implications for exploring modifiable risk factors for social disconnection and for understanding the mechanisms linking social connection to health-related outcomes.

## Key Points

**Question:**

Are there any distinct patterns of how people are socially connected and disconnected, and if so, how are those patterns associated with health and well-being outcomes?

**Findings:**

In this cohort study of 7706 participants aged 50 years or older, 5 clusters of social connection were identified using machine learning cluster analysis. The analysis found that deficits in any of the structural, functional, or quality dimensions of social connection were associated with poorer outcomes.

**Meaning:**

These findings suggest that it is important to adopt a holistic approach of measuring social connection and to understand its heterogenous patterns.

## Teaser

This cohort study identifies patterns of social connection and evaluates whether they are associated with health and well-being outcomes among older adults in England.

## Introduction

The last few decades have seen a growing awareness of the importance of social connection, with many empirical studies showing their impacts on longevity as well as various health and well-being outcomes.^1,2,3,4,5^ This growing interest is also manifested in national and global policies and programs.^6,7^ For example, the United Kingdom launched a national loneliness strategy and appointed its first minister for loneliness in 2018,^8^ and in 2023, the World Health Organization launched the Commission on Social Connection (2024-2026), recognizing this issue as a global public health priority.^9^

Social connection can be broadly defined as the extent to which an individual is engaged physically, behaviorally, and emotionally with other people.^10,11^ It is generally agreed that social connection is a multidimensional construct, encompassing a number of related but distinct concepts, such as loneliness, social isolation, social support, one’s social network, social integration, and so forth. Different frameworks have been proposed to understand the dimensionality of social connection. For example, social connection can be divided into objective (eg, frequency of social contact, living alone) vs subjective (eg, loneliness, relationship strain) dimensions^12^ or social assets (eg, social support) vs deficits (eg, social isolation).^10^ Other models categorize social connection in 3 dimensions, with a structural dimension including many aspects of the objective dimension, a functional dimension (emphasizing the support that we receive from our social connections to meet specific needs) combining subjective and objective components, and a quality dimension (assessing positive and negative affects) largely focused on subjective assessments.^11^

The existence of this multitude of different social connection frameworks has led to 3 key research challenges. First, it is unclear whether different individuals display differential patterns of social connection, as previously hypothesized.^13^ Second, it is unclear whether any differences in these patterns are associated with health-related outcomes. In other words, are some patterns of social connection more strongly linked to morbidity or mortality than others? Third, given a lack of consensus about the empirical validity of one theoretical model over another, most empirical studies focused on a single or few individual concepts of social connection, rather than understanding in full detail the interrelationships between social connection components and their health impacts.^14,15,16^ As a result, the existing empirical evidence on social connection and its health impact is generally piecemeal and fragmented.

Therefore, this study aimed to provide a data-driven approach to understand social connection. Using machine learning approaches in a large representative cohort of older adults in England, we sought to identify clusters of people with similar patterns of social connection across different individual concepts and to break away from the conventional measurement approach by exploring a typology of qualitative categories. We then aimed to assess whether a novel data-driven model of social connection led to new insight into the association of social connection with different outcomes, including mental health, well-being, general health, and health behavior.

## Methods

### Data

Data were from the English Longitudinal Study of Aging (ELSA). ELSA is a nationally representative longitudinal study of people aged 50 years and older and their partners living in private households in England. The original sample was drawn from individuals who participated in the Health Survey in England (HSE). The first wave of data collection commenced in 2002 to 2003. Participants are followed up biennially, with refreshment samples added in later waves. This study used data from wave 4 (2008-2009) and wave 5 (2010-2011). Wave 4 was treated as the baseline, as some measures (volunteering) were not measured properly in previous waves. We excluded participants who did not return self-completion questionnaires where most social connection measures were collected. After excluding participants with missing data on any of these measures, we had an analytical sample of 7706 participants for the cluster analysis. Further regression analysis excluded participants with missing self-completion survey weights, providing a subsample of 6983 participants for regression analyses. eFigure 1 in Supplement 1 presents the sample selection diagram. Ethical approval for ELSA was granted by the Berkshire Research Ethics Committee. All participants gave informed consent. This study followed the Strengthening the Reporting of Observational Studies in Epidemiology (STROBE) reporting guidelines.

### Measurements

#### Social Connection Indicators

We identified 8 variables within ELSA that measure different dimensions of social connection, including living alone (yes vs no); network diversity, defined as the number of different types of social relationships (partner, children, friends, relatives); network size, defined as the number of close relationships; social integration, measured by organizational membership and volunteering; social isolation, measured by contacts with children, friends, and relatives; loneliness, measured by the 3-item UCLA loneliness scale; and positive (satisfaction) and negative (strain) aspects of relationships with partner, children, friends, and relatives. Table 1 shows how these factors group according to 2 of the dominant theoretical models of social connection. Living alone and network diversity were treated as categorical variables. The rest were continuous, which were standardized to have a mean of 0 and an SD of 1.

**Table 1.** Components and Indicators of Social Connection

Subjective-objective model	Indicators	Questions
**Structural**		
Objective	Living alone	Number of people in the household
Objective	Social network diversity	Have a partner Have any child Have any relative Have any friend
Subjective	Intimate network size	How close is your relationship with your spouse or partner? How many of your children would you say you have a close relationship with? How many of these family members would you say you have a close relationship with? How many of your friends would you say you have a close relationship with?
Objective	Social integration	Are you a member of any organizations, clubs, or societies? Thinking about all the organizations, clubs, or societies that you are a member of, how many committee meetings, if any, do you attend in a year? In the last 12 mo, have you given any unpaid help to any groups, clubs, or organizations? Have you given any unpaid help to other people, such as a friend, neighbor, or someone else, but not a relative?
Objective	Social isolation^a^	How often do you meet up with your partner, children, relatives, and friends? How often do you speak on the phone with your partner, children, relatives, and friends?
**Functional**		
Subjective	Loneliness	How often do you feel you lack companionship? How often do you feel left out? How often do you feel isolated from others?
**Quality**		
Subjective	Positive aspects and satisfactions^a^	How much do your partner, children, relatives, or friends really understand the way you feel about things? How much can you rely on your partner, children, relatives, or friends if you have a serious problem? How much can you open up to your partner, children, relatives, or friends if you need to talk about your worries?
Subjective	Negative aspects and strains^a^	How much do your partner, children, relatives, or friends criticize you? How much do your partner, children, relatives, or friends let you down when you are counting on them? How much do your partner, children, relatives, or friends get on your nerves?

^a^ Participants were asked about this aspect of their relationships with each component of their social network (ie, partner, children, relatives, and friends) separately.

### Outcomes and Covariates

We considered 6 outcomes related to mental health, hedonic and eudaimonic well-being, general health, and health behavior. First, depressive symptoms were measured on the 8-item Center for Epidemiologic Studies Depression Scale, ranging from 0 to 8. We used a score of 3 or greater for depression.^17^ Second, life satisfaction was measured using the Satisfaction With Life Scale,^18^ with a higher value indicating a higher level of satisfaction. Third, pleasure was assessed with the subscale of the CASP19 quality of life scale,^19^ with a higher value indicating a higher level of pleasure. Fourth, self-realization was also assessed with the CASP19 subscale.^19^ Fifth, self-reported health was originally measured on a 5-point scale but was recoded as a binary variable of having excellent, very good, or good health vs fair or poor health. Sixth, physical activity was coded as a binary variable of weekly moderate or vigorous physical activity.^20^

Using directed acyclic graphs, we identified a number of key confounders, including age (<60, 60-69, 70-79, and ≥80 years), sex (male and female), ethnicity (Asian, Asian British, Black, Black British, mixed ethnic group, White, and any other group; dichotomized as White and other), education (no qualification, A level [equivalent to a year 13 high school certificate] or below, and higher education), social class based on the National Statistics Socio-Economic Classification (low, medium, and high), and wealth quintiles. Ethnicity was dichotomized given the small number of individuals identifying as Asian, Asian British, Black, Black British, mixed ethnic group, or any other ethnic group. eTable 1 in Supplement 1 provides descriptive statistics of all variables. eTable 2 in Supplement 1 shows the correlation matrix of social connection indicators.

### Statistical Analysis

This study used a model-based clustering algorithm, KAMILA, short for Kay-Means for Mixed Large Data.^21,22^ It clusters continuous variables like the K-means algorithm and categorical variables based on Gaussian-multinomial mixture models. We applied the KAMILA algorithm with different numbers of clusters. The optimal number of clusters was chosen based on the prediction strength method.^23^ We defined the optimal number of clusters as the largest number of clusters with a prediction strength greater than the threshold of 0.8.^21^

Sensitivity analyses were carried out using the k-prototypes algorithm using Gower distance via R package clustMixTy^24^ and Gaussian mixture model clustering method via R package VarSelLCM.^25^ These algorithms were chosen because they have been shown to outperform other alternatives for mixed data of categorical and continuous variables.^26^

To test the association of the newly generated clustering variable with health-related outcomes, we fitted linear and logistic regression models on continuous and binary outcome measures, respectively. All independent variables were taken from wave 4 and outcome measures from wave 5. For each outcome, we fitted 2 sets of models with and without controlling for baseline (wave 4) outcome. We acknowledge that baseline adjustment is controversial, which may lead to downward bias in presence of residual autocorrelation,^27^ while leaving it out puts us at risk of omitted variable bias. By presenting 2 sets of results, we hope to posit plausible bounds on the true association between the social connection cluster and outcome. Clustering analysis was unweighted, but the regression analyses were weighted using self-completion weights from wave 4. Missing data for regression analyses were dealt with using multiple imputation by chained equations, using classification and regression trees, with 30 imputations and 10 iterations. We applied the Benjamini and Hochberg procedure to correct for multiple testing by reporting adjusted *P* values, which are reported as a continuous measure without any threshold for statistical significance.^28^ All analyses were implemented in R version 4.3.3 (R Project for Statistical Computing).

## Results

### Cluster Analysis

**Figure 1.**
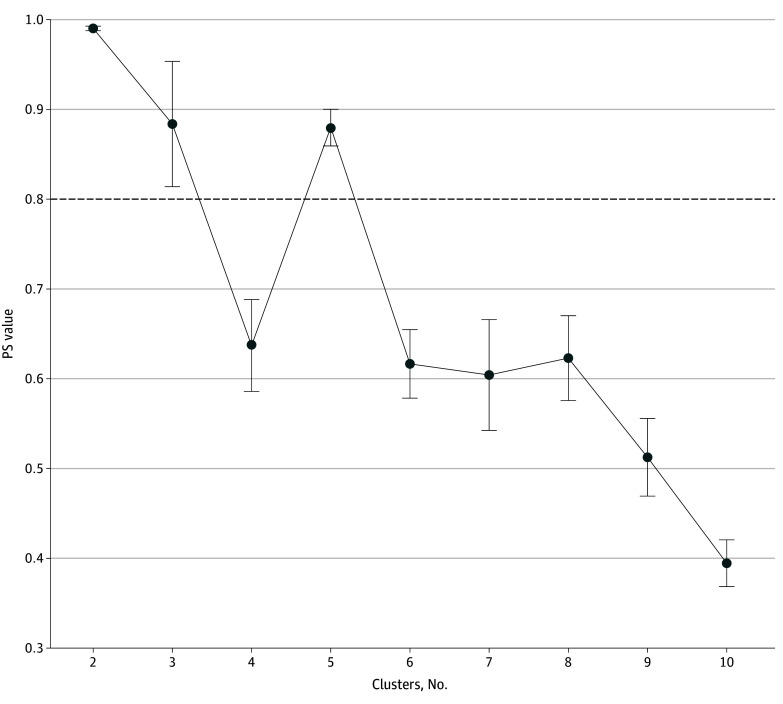
Prediction Strength (PS) by the Number of Clusters The dashed horizontal line indicates a PS threshold of 0.8.

**Figure 2.**
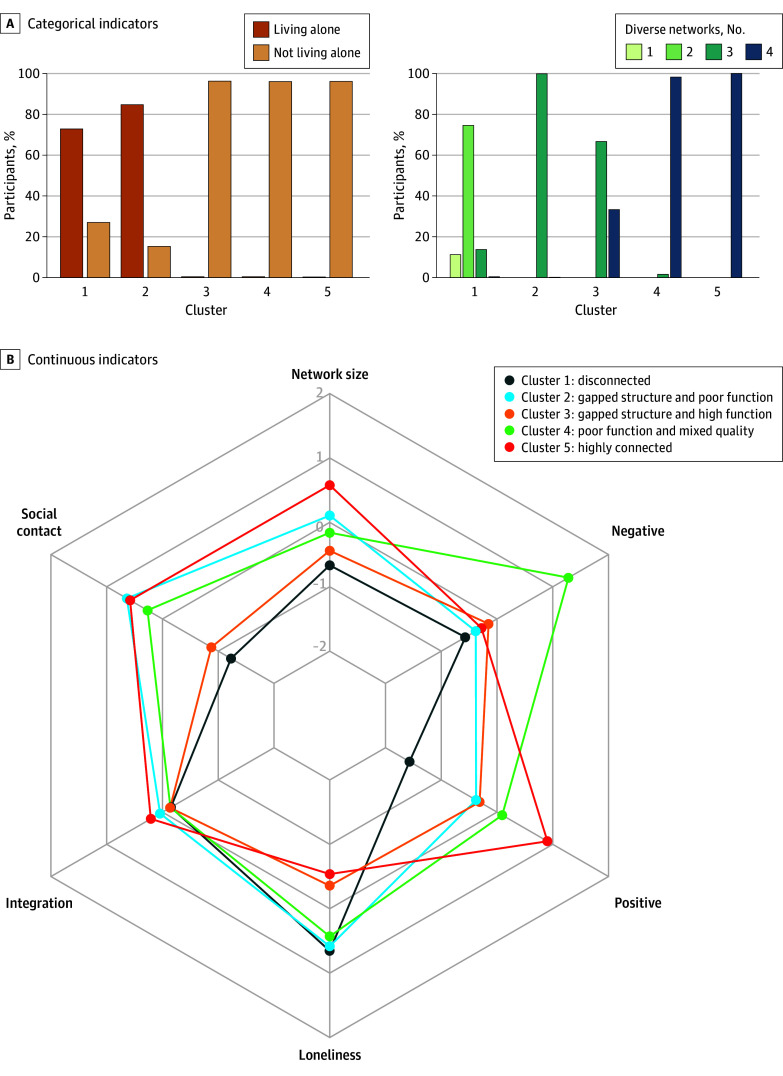
Cluster Profiles

Among 7706 participants (mean [SD] age, 64.7 [9.6] years; 4248 [55.1%] female; 7536 [97.8%] White) in the cluster analysis, 1674 (21.7%) lived alone and 4558 (59.1%) reported having a diverse network that consisted of partner, children, relatives, and friends (eTable 1 in Supplement 1). The analytical sample had a mean (SD) network size of 8.12 (4.57) and mean (SD) loneliness score of 4.18 (1.54) on the unstandardized scale. As shown in Figure 1, the prediction strength method indicated that 5 was the optimal number of clusters. Figure 2 shows the summary statistics of both categorical and continuous indicators by cluster. Table 2 presents the features of the 5 clusters.

**Table 2.** Summary of the Social Connection Features of the 5 Identified Clusters

Cluster	Participants, No. (%)	Holt-Lunstad model			Assessment	
		Structure	Function	Quality	Objective	Subjective
1: Disconnected	974 (12.6)	Poor	Poor	Poor	Poor	Poor
2: Gapped structure/poor function	1109 (14.4)	Gapped	Poor	Medium	Medium	Poor
3: Gapped structure/high function	1582 (20.5)	Gapped	High	Medium	Medium	Medium
4: Poor function/mixed quality	1501 (19.5)	High	Poor	Mixed	High	Poor
5: Highly connected	2540 (33.0)	High	High	High	High	High

Cluster 5 (highly connected) was the largest in the sample (2540 [33.0%]), which was characterized by strong structural social connection (S_1_), high function (F_1_), and high quality (Q_1_). More specifically, older adults in this cluster were almost exclusively living with others and had a diverse social network, a large network size, and frequent social contacts. They also had low levels of loneliness and negative affect and a high level of positive affect.

In contrast, cluster 1 (disconnected; 974 [12.6%]) was characterized by weak structural social connection (S_0_; a higher probability of living alone, low network diversity, small network size, and infrequent social contacts); poor function (F_0_), indicated by a high level of loneliness; and poor quality (Q_0_), indicated by a low level of positive affect.

Cluster 2 (gapped structure/poor function; 1109 [14.4%]) was characterized by having gaps in structural measures (S_g_; with a high probability of living alone and low network diversity but relatively large network size and frequent social contacts). It had a decent level of quality (Q_1_), but poor function (F_0_; high level of loneliness).

Cluster 3 (gapped structure/high function; 1582 [20.5%]) consisted of people living with others, with moderately diverse social network (S_g_). However, individuals in this group had gaps in other structural measures, particularly network size and social contact. Social connection seemed to be highly functional (F_1_) and of decent quality (Q_1_).

Cluster 4 (poor function/mixed quality; 1501 [19.5%]) had relatively strong structural social connection (S_1_). However, it was characterized by poor function (F_0_; high level of loneliness) and mixed quality (Q_0_; high level of negative affect and relatively high level of positive affect) (Figure 2).

Sensitivity analyses using the k-prototype method and Gaussian mixture model supported the 5-cluster solution (eFigure 2 in Supplement 1). The k-prototype method gave largely consistent cluster partitions as KAMILA (eTable 3 and eFigure 3 in Supplement 1), but the Gaussian mixture model had a poor agreement with either KAMILA or k-prototype (eTable 3 and eFigure 4 in Supplement 1), which was likely because the multivariate normal assumption was violated (eTable 4 in Supplement 1). It has been previously suggested KAMILA is more tolerant of variable distributions and offers the best performance in case of large data.^26^

### Clusters and Health Outcomes

Among 6983 participants (mean [SD] age, 65.5 [9.2] years) in the regression analysis, 3833 (54.9%) were female participants and 3150 (45.1%) male participants (eTable 1 in the Supplement). Table 3 reports the results from regression models on each outcome measure with and without controlling for baseline outcome. Compared with the highly connected cluster (cluster 5; S_1_F_1_Q_1_), all other clusters were more likely to have poorer outcomes. For example, individuals in the disconnected cluster (cluster 1; S_0_F_0_Q_0_) were more than twice as likely as those in the highly connected cluster to have depression (odds ratio [OR], 2.73; 95% CI, 2.24 to 3.33). Most of these differences persisted after controlling for baseline outcome (eg, depression for cluster 1 vs 5: OR, 1.95; 95% CI, 1.57 to 2.43). For most outcomes, the magnitude of the difference with the highly connected cluster was the smallest for the gapped structure/high function cluster (cluster 3; S_g_F_1_Q_1_) (eg, depression for cluster 3 vs 5: OR, 1.34; 95% CI, 1.10-1.64; after controlling for baseline outcome: OR, 1.28; 95% CI, 1.03-1.59). There was limited evidence that the disconnected cluster was different from other clusters except for the highly connected cluster on self-reported health and physical activity (eTable 5 in Supplement 1). But differences were found in mental health and well-being outcomes. The gapped structure/poor function cluster (cluster 2; S_g_F_0_Q_1_) and particularly the poor function/mixed quality cluster (cluster 4; S_1_F_0_Q_0_) were more similar to the disconnected cluster than the other 2 clusters in terms of their associations with the outcome measures. eFigure 4 in Supplement 1 shows the residual diagnoses for linear regressions.

**Table 3.** Results From Regression Analysis With 6983 Participants and 30 Imputations^a^

Outcomes	Model 1			Model 2		
	OR (95% CI)	*P* value	BH-adjusted *P* value	OR (95% CI)	*P* value	BH-adjusted *P* value
**Depression**						
1 vs 5 (S_0_F_0_Q_0_ vs S_1_F_1_Q_1_)	2.73 (2.24 to 3.33)	<.001	<.001	1.95 (1.57 to 2.43)	<.001	<.001
2 vs 5 (S_g_F_0_Q_1_ vs S_1_F_1_Q_1_)	2.22 (1.81 to 2.71)	<.001	<.001	1.55 (1.24 to 1.94)	<.001	<.001
3 vs 5 (S_g_F_1_Q_1_ vs S_1_F_1_Q_1_)	1.34 (1.10 to 1.64)	.004	.009	1.28 (1.03 to 1.59)	.02	.05
4 vs 5 (S_1_F_0_Q_0_ vs S_1_F_1_Q_1_)	2.18 (1.79 to 2.66)	<.001	<.001	1.62 (1.30 to 2.02)	<.001	<.001
**Life satisfaction, coefficient (95% CI)**						
1 vs 5 (S_0_F_0_Q_0_ vs S_1_F_1_Q_1_)	−5.12 (−5.65 to −4.59)	<.001	<.001	−0.96 (−1.39 to −0.54)	<.001	<.001
2 vs 5 (S_g_F_0_Q_1_ vs S_1_F_1_Q_1_)	−3.55 (−4.07 to −3.03)	<.001	<.001	−0.34 (−0.75 to 0.07)	.10	.13
3 vs 5 (S_g_F_1_Q_1_ vs S_1_F_1_Q_1_)	−1.98 (−2.43 to −1.53)	<.001	<.001	−0.54 (−0.88 to −0.19)	.002	.005
4 vs 5 (S_1_F_0_Q_0_ vs S_1_F_1_Q_1_)	−3.55 (−4.01 to −3.09)	<.001	<.001	−0.76 (−1.13 to −0.39)	<.001	<.001
**Quality of life, pleasure, coefficient (95% CI)**						
1 vs 5 (S_0_F_0_Q_0_ vs S_1_F_1_Q_1_)	−1.91 (−2.10 to −1.72)	<.001	<.001	−0.53 (−0.68 to −0.37)	<.001	<.001
2 vs 5 (S_g_F_0_Q_1_ vs S_1_F_1_Q_1_)	−1.04 (−1.22 to −0.85)	<.001	<.001	−0.27 (−0.42 to −0.11)	.001	.002
3 vs 5 (S_g_F_1_Q_1_ vs S_1_F_1_Q_1_)	−0.87 (−1.04 to −0.71)	<.001	<.001	−0.29 (−0.42 to −0.15)	<.001	<.001
4 vs 5 (S_1_F_0_Q_0_ vs S_1_F_1_Q_1_)	−1.33 (−1.50 to −1.16)	<.001	<.001	−0.35 (−0.48 to −0.21)	<.001	<.001
**Quality of life, self-realization, coefficient (95% CI)**						
1 vs 5 (S_0_F_0_Q_0_ vs S_1_F_1_Q_1_)	−2.13 (−2.39 to −1.88)	<.001	<.001	−0.44 (−0.64 to −0.24)	<.001	<.001
2 vs 5 (S_g_F_0_Q_1_ vs S_1_F_1_Q_1_)	−1.41 (−1.65 to −1.16)	<.001	<.001	−0.29 (−0.48 to −0.10)	.003	.007
3 vs 5 (S_g_F_1_Q_1_ vs S_1_F_1_Q_1_)	−0.92 (−1.13 to −0.70)	<.001	<.001	−0.18 (−0.34 to −0.01)	.04	.07
4 vs 5 (S_1_F_0_Q_0_ vs S_1_F_1_Q_1_)	−1.66 (−1.87 to −1.44)	<.001	<.001	−0.40 (−0.57 to −0.23)	<.001	<.001
**Self-reported health**						
1 vs 5 (S_0_F_0_Q_0_ vs S_1_F_1_Q_1_)	0.57 (0.47 to 0.69)	<.001	<.001	0.72 (0.56 to 0.91)	.005	.01
2 vs 5 (S_g_F_0_Q_1_ vs S_1_F_1_Q_1_)	0.70 (0.58 to 0.85)	<.001	<.001	0.77 (0.62 to 0.97)	.03	.04
3 vs 5 (S_g_F_1_Q_1_ vs S_1_F_1_Q_1_)	0.67 (0.55 to 0.80)	<.001	<.001	0.65 (0.53 to 0.81)	<.001	<.001
4 vs 5 (S_1_F_0_Q_0_ vs S_1_F_1_Q_1_)	0.57 (0.48 to 0.69)	<.001	<.001	0.71 (0.57 to 0.87)	.001	.003
**Physical activity**						
1 vs 5 (S_0_F_0_Q_0_ vs S_1_F_1_Q_1_)	0.65 (0.53 to 0.80)	<.001	<.001	0.75 (0.60 to 0.95)	.02	.04
2 vs 5 (S_g_F_0_Q_1_ vs S_1_F_1_Q_1_)	0.70 (0.57 to 0.85)	<.001	.001	0.75 (0.60 to 0.94)	.01	.03
3 vs 5 (S_g_F_1_Q_1_ vs S_1_F_1_Q_1_)	0.79 (0.65 to 0.96)	.02	.028	0.83 (0.67 to 1.03)	.10	.15
4 vs 5 (S_1_F_0_Q_0_ vs S_1_F_1_Q_1_)	0.71 (0.58 to 0.87)	.001	<.001	0.79 (0.63 to 0.99)	.04	.07

Abbreviations: BH, Benjamini and Hochberg; F_0_, poor function; F_1_, high function; OR, odds ratio; Q_0_, low quality; Q_1_, high quality; S_0_, poor structure; S_1_, high structure; S_g_, gapped structure.

^a^ Model 1 controlled for all covariates, including age, gender, ethnicity, education, social class, and wealth; model 2 additionally controlled for the corresponding outcome measure at baseline (wave 4).

## Discussion

To our knowledge, the present study is among the first studies to explore the possibility of heterogeneous patterns of social connection using empirical data. Based on cluster analysis, we identified 5 clusters of social connection across a diverse set of indicators (Table 2).

These findings shed light on existing theoretical models. In relation to Holt-Lunstad’s social connection framework,^11^ we did indeed see differentiation of social connection across assessments of structure, function, and quality. In particular, our data-driven approach supports previous assertions that the presence of weak structural connections is not found alongside strong function or quality, so if a structural foundation is absent, there may be little or no opportunity to experience function or quality components. However, a solid structural foundation is not a guarantee to relationship function or quality, and an all-round structure is not necessary for high function and high quality. It is possible to draw sufficient support from a narrow structural foundation to meet one’s needs.^13^ Four of the identified clusters are perfectly in line with the hypothetical examples in an early work.^13^ The only exception is the cluster with strong structural foundation but poor function and mixed quality (cluster 4), which was not previously hypothesized. This cluster could include individuals who are in complicated relationships, having a partner and family and friends around them but not receiving sufficient social support, feeling lonely, and experiencing both high positivity and negativity in their relationships. The fact that this population has not been identified theoretically previously but is evident in this data-driven approach highlights the importance of future research. We also identified that there was major variation across the clusters as to the strength of objective vs subjective assessments of social connection. Many studies have used just single items of objective vs subjective social connection, such as social isolation vs loneliness. But our clusters highlight that there are many more variations of objective and subjective connection patterns, underscoring the importance of using multidimensional instead of unidimensional measures.

A key question that follows is whether the profile differences between clusters are associated with any material difference in health-related outcomes. We addressed this question by fitting a series of regression models on a number of outcomes. Overall, our analyses provided strong and consistent evidence that all clusters have poorer outcomes compared with the highly connected cluster, including the gapped structure/high function cluster, which arguably has the closest resemblance to the highly connected cluster but falls short of some structural measures and level of positive affect. This suggests that any deficit in each of the social connection components and even individual indicators within each component could lead to negative outcomes and is consistent with existing theoretical and empirical research on the relationship between social connection and health outcomes,^1,2,3,4,5^ serving as an external validation of the cluster analysis. But we were also able to probe specific hypotheses further. Holt-Lunstad and Steptoe^13^ have previously theorized that if individuals have gaps in the structural foundations of their social connections, this can lead to greater vulnerability in function or quality. Two of our clusters had gapped structure, but while cluster 2 had poor function, cluster 3 had high function. Interestingly, both clusters reported similar quality results. This enabled us to explore the specific differences in outcomes between these 2 groups and assess the relative importance of function. Notably, the odds of poorer outcomes were not statistically significantly better in cluster 3 (high function) compared with cluster 2 (low function), suggesting that adequate quality may have been protective. Indeed, cluster 4 had weak function (like cluster 2) but had strong structure, which might have led to hypotheses of better outcomes. But it arguably had the second worst outcomes (after cluster 1). This result is most logically attributable to the quality component, which (despite having higher levels of positive affect) also had much higher levels of negative affect. Taking an alternative theoretical angle, it was the weakness in the subjective rather than objective measures that was most problematic. This is in line with studies highlighting the impact of negative experiences in relationship quality on health outcomes.^29^ However, future research is needed to explore these findings further to assess whether gaps or weaknesses in some aspects of structure or function or quality (eg, living alone) are more important than others.

In the existing literature, many potential mechanisms have been proposed to explain the association between selected measures of social connection and health-related outcomes, including psychological, behavioral, and biological pathways.^30,31,32,33,34,35^ Different mechanisms may come into play for different outcomes, which may explain why differential associations with the cluster variable were found for different outcomes. Further research is needed to understand the nuance of this, in particular when considering different patterns of social connection that individuals may experience, as presented here. Such work could provide greater insight into the relationship between social connection and complex health outcomes. Further work is also needed to understand the formation and dynamics of different patterns of social connection and whether different combinations trigger multiple mechanisms, having additive or multiplicative health impacts.

### Strengths and Limitations

This study has strengths. It is important to acknowledge that there exists early work showing patterns of social connection.^14,15,16^ However, these studies considered only a limited number of indicators, mostly related to loneliness and social isolation, omitting relationship quality measures and some important structural measures, such as network size and diversity. The present study expands the existing research by selecting a much wider range of indicators that can be mapped to well-established conceptual frameworks.^10,11,12^ Furthermore, existing research used either latent class or latent profile analysis, which are restricted to either categorical or continuous variables. Our study has the advantage of using machine learning algorithms that are well suited to handle mixed data. And our findings have been validated by using 3 different algorithms. Other strengths of the present study include using a nationally representative sample and rigorous analyses accounting for self-selection bias by weighting and missing data bias by multiple imputation.

However, our study is not without limitations. First, our data were drawn from older adults in England. It is unclear whether these findings can be extrapolated to other age groups or societal contexts. Second, although weights were applied in regression analyses, it should be noted that cluster analyses were unweighted, for which we cannot rule out the possibility of self-selection bias. Third, despite the richness of data, our list of social connection indicators is by no means exhaustive. For example, there was no measure of received or perceived social support in the functional dimension or measure of social inclusion or exclusion in the quality dimension. Therefore, future studies on different populations, with a full list of social connection indicators are recommended. Additionally, ethnicity was used as a binary variable because of the small number of participants from ethnic minority backgrounds. Future work is needed to investigate the influence of racial and ethnic identities as well as associated racism and cultural caste systems.

## Conclusions

In this cohort study of older adults in England, we applied novel machine learning approaches, providing a data-driven classification of social connection. Our findings validated and extended some previous theoretical assumptions about patterns of social connection but also demonstrated how using conventional measurement approaches (ordinal or scale measurements) or taking individual measures of specific aspects of social connection out of context poses challenges. Our analyses found that a deficit in the structure, function, or quality of social connection was associated with poorer health-related outcomes. But poor relationship quality, even in the presence of strong relationship function and decent structure, was especially problematic. Overall, this study highlighted the importance of adopting a multidimensional measurement approach of social connection and understanding the nuance of heterogenous patterns of social connection. Understanding the typologies of social connection has significant implications for exploring modifiable risk factors for social disconnection and for understanding the mechanisms linking social connection to health-related outcomes.

## Notes

Supplement 1. **eTable 1.** Unstandardized and Unweighted Descriptive Statistics **eTable 2.** Correlation Matrix of Social Connection Indicators **eTable 3.** Comparing Cluster Memberships Based on KAMILA and K-Prototypes Algorithms **eTable 4.** Test Results of Multivariate Normal Assumption **eTable 5.** Results From Regression Analysis Using Cluster 1 as the Reference **eTable 6.** Results From Regression Analysis Using Cluster 2 as the Reference **eFigure 2.** Total Within-Clusters Sum of Squares by the Number of Clusters Using the K-Prototypes Algorithm and Integrated Completed Likelihood (ICL) Criterion Using Gaussian Mixture Model **eFigure 3.** Cluster Profiles Using the K-Prototypes Algorithm **eFigure 4.** Cluster Profiles Using Latent Class Model **eFigure 5.** Linear Regression Residual Diagnostics

Supplement 2.
Data Sharing Statement
