# Supplementary material for: Patterns of Social Connection Among Older Adults in England
Source: JAMA Netw Open. 2024 Dec 23;7(12):e2451580. doi: 10.1001/jamanetworkopen.2024.51580 (PMC11667348; doi:10.1001/jamanetworkopen.2024.51580)
Supplement: Supplement 1. — eTable 1. Unstandardized and Unweighted Descriptive Statistics eTable 2. Correlation Matrix of Social Connection Indicators eTable 3. Comparing Cluster Memberships Based on KAMILA and K-Prototypes Algorithms eTable 4. Test Results of Multivariate Normal Assumption eTable 5. Results From Regression Analysis Using Cluster 1 as the Reference eTable 6. Results From Regression Analysis Using Cluster 2 as the Reference eFigure 2. Total Within-Clusters Sum of Squares by the Number of Clusters Using the K-Prototypes Algorithm and Integrated Completed Likelihood (ICL) Criterion Using Gaussian Mixture Model eFigure 3. Cluster Profiles Using the K-Prototypes Algorithm eFigure 4. Cluster Profiles Using Latent Class Model eFigure 5. Linear Regression Residual Diagnostics [file jamanetwopen-e2451580-s001.pdf]

Supplemental Online Content

Bu F, Fancourt D. Patterns of social connection among older adults in England. *JAMA Netw Open.* 2024;7(12):e2451580. doi:10.1001/jamanetworkopen.2024.51580

eTable 1. Unstandardized and Unweighted Descriptive Statistics

eTable 2. Correlation Matrix of Social Connection Indicators

eTable 3. Comparing Cluster Memberships Based on KAMILA and K-Prototypes Algorithms

eTable 4. Test Results of Multivariate Normal Assumption

eTable 5. Results From Regression Analysis Using Cluster 1 as the Reference

eTable 6. Results From Regression Analysis Using Cluster 2 as the Reference

eFigure 2. Total Within-Clusters Sum of Squares by the Number of Clusters Using the K-Prototypes Algorithm and Integrated Completed Likelihood (ICL) Criterion Using Gaussian Mixture Model

eFigure 3. Cluster Profiles Using the K-Prototypes Algorithm

eFigure 4. Cluster Profiles Using Latent Class Model

eFigure 5. Linear Regression Residual Diagnostics

This supplemental material has been provided by the authors to give readers additional information about their work.

eTable 1. Unstandardized and Unweighted Descriptive Statistics

Variables: cluster analytical sample (n=7,706)	%/mean (SD)	Min-max	% missing data
Living alone: yes	21.7%	--	--
Living alone: no	78.3%	--	--
Network diversity: 1	1.4%	--	--
Network diversity: 2	9.4%	--	--
Network diversity: 3	30.0%	--	--
Network diversity: 4	59.1%	--	--
Network size	8.12 (4.57)	0-30	--
Social contact	18.53 (5.60)	0-30	--
Social integration	2.85 (2.34)	0-8	--
Loneliness	4.18 (1.54)	3-9	--
Negative aspects	23.06 (7.19)	0-36	--
Positive aspects	7.02 (4.88)	0-30	--
**Variables: regression analytical sample (n=6,983)**	**%/mean (SD)**	**Min-max**	**% missing data**
(Wave 4) Depression: <3	80.2%	--	0.6%
(Wave 4) Depression: ≥3	19.8%	--	0.6%
(Wave 4) Life satisfaction	25.35 (6.23)	5-35	2.2%
(Wave 4) Quality of life: pleasure	13.14 (2.29)	0-15	0.7%
(Wave 4) Quality of life: self-realisation	9.95 (3.10)	0-15	0.7%
(Wave 4) Self-reported health: Fair/poor	24.1%	--	0.0%
(Wave 4) Self-reported health: good/very good/excellent	75.9%	--	0.0%
(Wave 4) Physical activity: less than weekly	21.0%	--	0.0%
(Wave 4) Physical activity: weekly exercise	79.0%	--	0.0%
(Wave 5) Depression: <3	78.9%	--	12.7%
(Wave 5) Depression: ≥3	21.1%	--	12.7%
(Wave 5) Life satisfaction	25.81 (6.33)	5-35	17.6%
(Wave 5) Quality of life: pleasure	13.14 (2.28)	0-15	16.6%
(Wave 5) Quality of life: self-realisation	9.98 (3.14)	0-15	16.6%
(Wave 5) Self-reported health: Fair/poor	24.2%	--	11.8%
(Wave 5) Self-reported health: good/very good/excellent	75.8%	--	11.8%
(Wave 5) Physical activity: less than weekly	22.1%	--	10.7%
(Wave 5) Physical activity: weekly exercise	77.9%	--	10.7%
Age <60	29.5%	--	0.0%
Age 60-69 (vs <60)	38.9%	--	0.0%
Age 70-79 (vs <60)	23.3%	--	0.0%
Age 80+ (vs <60)	8.3%	--	0.0%
Sex: female	54.9%	--	0.0%
Sex: male	45.1%	--	0.0%
Ethnicity: white	97.9%	--	0.0%
Ethnicity: other	2.1%	--	0.0%
Education: high	32.9%	--	0.0%
Education: medium	41.4%	--	0.0%
Education: low	25.7%	--	0.0%

© 2024 Bu F et al. *JAMA Network Open.*

Social class: high	35.4%	--	1.8%
Social class: medium	25.8%	--	1.8%
Social class: low	38.8%	--	1.8%
Wealth quintile: 1	15.3%	--	1.9%
Wealth quintile: 2	19.2%	--	1.9%
Wealth quintile: 3	20.0%	--	1.9%
Wealth quintile: 4	21.7%	--	1.9%
Wealth quintile: 5	23.8%	--	1.9%

© 2024 Bu F et al. *JAMA Network Open.*

eTable 2. Correlation Matrix of Social Connection Indicators (N=9,064)

	Living alone	Network diversity	Network size	Social contact	Social integration	Loneliness	Positive affect	Negative affect
Living alone	1.00	-0.65	-0.11	-0.05	-0.01	0.30	-0.46	-0.26
Network diversity	-0.65	1.00	0.27	0.49	0.05	-0.26	0.70	0.31
Network size	-0.11	0.27	1.00	0.40	0.15	-0.19	0.44	-0.05
Social contact	-0.05	0.49	0.40	1.00	0.09	-0.11	0.55	0.08
Social integration	-0.01	0.05	0.15	0.09	1.00	-0.10	0.10	0.00
Loneliness	0.30	-0.26	-0.19	-0.11	-0.10	1.00	-0.41	0.17
Positive affect	-0.46	0.70	0.44	0.55	0.10	-0.41	1.00	0.01
Negative affect	-0.26	0.31	-0.05	0.08	0.00	0.17	0.01	1.00

Notes: Point-biserial correlation was used to measure the association of living alone (binary) with other variables. The rest was assessed by Pearson correlation. Correlation strength: 0-0.19 very weak, 0.2-0.39 weak, 0.4-0.59 moderate, 0.6-0.79 strong, 0.8-1 very strong

© 2024 Bu F et al. *JAMA Network Open.*

eTable 3. Comparing Cluster Memberships Based on KAMILA and K-Prototypes Algorithms

		K-prototype						
		Cluster 1	Cluster 2	Cluster 3	Cluster 4	Cluster 5	N	ARI
KAMILA	Cluster 1	14.6%	0.7%	0.0%	8.2%	76.5%	974	0.62
	Cluster 2	15.0%	0.0%	0.0%	84.9%	0.1%	1,109	
	Cluster 3	65.8%	25.0%	8.8%	0.4%	0.0%	1,582	
	Cluster 4	1.5%	83.5%	15.0%	0.0%	0.0%	1,501	
	Cluster 5	0.2%	9.4%	90.4%	0.0%	0.0%	2,540	
		Model-based clustering						
		Cluster 1	Cluster 2	Cluster 3	Cluster 4	Cluster 5	N	ARI
KAMILA	Cluster 1	3.7%	3.0%	0.0%	26.8%	66.5%	974	0.26
	Cluster 2	65.7%	6.5%	0.0%	24.9%	2.9%	1,109	
	Cluster 3	1.7%	30.8%	3.0%	64.1%	0.4%	1,582	
	Cluster 4	0.1%	28.3%	46.4%	25.1%	0.0%	1,501	
	Cluster 5	0.0%	0.0%	22.9%	77.0%	0.0%	2,540	
		Model-based clustering						
		Cluster 1	Cluster 2	Cluster 3	Cluster 4	Cluster 5	N	ARI
K-prototype	Cluster 1	7.3%	34.0%	0.0%	54.3%	4.4%	1,377	0.27
	Cluster 2	0.2%	28.1%	44.4%	27.3%	0.1%	1,895	
	Cluster 3	0.0%	0.1%	18.2%	81.6%	0.0%	2,660	
	Cluster 4	64.0%	0.9%	0.0%	26.0%	9.1%	1,028	
	Cluster 5	4.4%	0.1%	0.0%	24.4%	71.0%	746	

Notes: ARI-Adjusted Rand Index which is a measure of similarity between two clustering, ranging from -1 to 1, with 1 indicating a perfect agreement.

© 2024 Bu F et al. *JAMA Network Open.*

eTable 4. Test Results of Multivariate Normal Assumption

Test	Statistic	P
Mardia: Skewness	14329.73	<0.001
Mardia: Kurtosis	33.26	<0.001
Henze-Zirkler	13.16	<0.001
Doornik-Hansen	30758.94	<0.001

© 2024 Bu F et al. *JAMA Network Open.*

eTable 5. Results From Regression Analysis Using Cluster 1 as the Reference (N=6,983, MI=30)

	Model 1				Model 2			
**Depression**	**OR**	**95% CI**	**p**	**p_{BH}**	**OR**	**95% CI**	**p**	**p_{BH}**
2 vs 1 ($S_gF_0Q_1$ vs $S_0F_0Q_0$)	0.81	[0.67,0.99]	0.036	0.064	0.79	[0.64,0.99]	0.041	0.098
3 vs 1 ($S_gF_1Q_1$ vs $S_0F_0Q_0$)	0.49	[0.40,0.61]	<0.001	<0.001	0.66	[0.52,0.83]	<0.001	0.002
4 vs 1 ($S_1F_0Q_0$ vs $S_0F_0Q_0$)	0.80	[0.65,0.98]	0.035	0.064	0.83	[0.66,1.05]	0.122	0.210
5 vs 1 ($S_1F_1Q_1$ vs $S_0F_0Q_0$)	0.37	[0.30,0.45]	<0.001	<0.001	0.51	[0.41,0.64]	<0.001	<0.001
**Life Satisfaction**	**Coef.**	**95% CI**	**p**	**p_{BH}**	**Coef.**	**95% CI**	**p**	**p_{BH}**
2 vs 1 ($S_gF_0Q_1$ vs $S_0F_0Q_0$)	1.57	[1.00,2.14]	<0.001	<0.001	0.62	[0.17,1.07]	0.007	0.018
3 vs 1 ($S_gF_1Q_1$ vs $S_0F_0Q_0$)	3.15	[2.57,3.72]	<0.001	<0.001	0.43	[-0.03,0.89]	0.069	0.094
4 vs 1 ($S_1F_0Q_0$ vs $S_0F_0Q_0$)	1.57	[1.00,2.15]	<0.001	<0.001	0.20	[-0.25,0.65]	0.387	0.490
5 vs 1 ($S_1F_1Q_1$ vs $S_0F_0Q_0$)	5.12	[4.59,5.65]	<0.001	<0.001	0.96	[0.54,1.39]	<0.001	<0.001
**Quality of life: pleasure**	**Coef.**	**95% CI**	**p**	**p_{BH}**	**Coef.**	**95% CI**	**p**	**p_{BH}**
2 vs 1 ($S_gF_0Q_1$ vs $S_0F_0Q_0$)	0.87	[0.66,1.09]	<0.001	<0.001	0.26	[0.08,0.44]	0.004	0.014
3 vs 1 ($S_gF_1Q_1$ vs $S_0F_0Q_0$)	1.03	[0.83,1.24]	<0.001	<0.001	0.24	[0.07,0.41]	0.004	0.014
4 vs 1 ($S_1F_0Q_0$ vs $S_0F_0Q_0$)	0.58	[0.38,0.78]	<0.001	<0.001	0.18	[0.02,0.34]	0.030	0.072
5 vs 1 ($S_1F_1Q_1$ vs $S_0F_0Q_0$)	1.91	[1.72,2.10]	<0.001	<0.001	0.53	[0.37,0.68]	<0.001	<0.001
**Quality of life: self-realisation**	**Coef.**	**95% CI**	**p**	**p_{BH}**	**Coef.**	**95% CI**	**p**	**p_{BH}**
2 vs 1 ($S_gF_0Q_1$ vs $S_0F_0Q_0$)	0.72	[0.44,1.00]	<0.001	<0.001	0.15	[-0.07,0.38]	0.171	0.271
3 vs 1 ($S_gF_1Q_1$ vs $S_0F_0Q_0$)	1.22	[0.94,1.49]	<0.001	<0.001	0.27	[0.05,0.48]	0.014	0.033
4 vs 1 ($S_1F_0Q_0$ vs $S_0F_0Q_0$)	0.48	[0.20,0.75]	0.001	0.001	0.04	[-0.17,0.26]	0.690	0.714
5 vs 1 ($S_1F_1Q_1$ vs $S_0F_0Q_0$)	2.13	[1.88,2.39]	<0.001	<0.001	0.44	[0.24,0.64]	<0.001	<0.001
**Self-reported health**	**OR**	**95% CI**	**p**	**p_{BH}**	**OR**	**95% CI**	**p**	**p_{BH}**
2 vs 1 ($S_gF_0Q_1$ vs $S_0F_0Q_0$)	1.23	[1.00,1.51]	0.045	0.058	1.08	[0.84,1.39]	0.540	0.641
3 vs 1 ($S_gF_1Q_1$ vs $S_0F_0Q_0$)	1.17	[0.96,1.44]	0.128	0.154	0.91	[0.71,1.17]	0.481	0.611
4 vs 1 ($S_1F_0Q_0$ vs $S_0F_0Q_0$)	1.01	[0.82,1.24]	0.925	0.925	0.99	[0.77,1.27]	0.913	0.913
5 vs 1 ($S_1F_1Q_1$ vs $S_0F_0Q_0$)	1.76	[1.45,2.15]	<0.001	<0.001	1.40	[1.10,1.77]	0.005	0.015
**Physical activity**	**OR**	**95% CI**	**p**	**p_{BH}**	**OR**	**95% CI**	**p**	**p_{BH}**
2 vs 1 ($S_gF_0Q_1$ vs $S_0F_0Q_0$)	1.07	[0.86,1.32]	0.547	0.579	1.00	[0.79,1.27]	0.996	0.996
3 vs 1 ($S_gF_1Q_1$ vs $S_0F_0Q_0$)	1.21	[0.97,1.50]	0.092	0.145	1.10	[0.86,1.40]	0.444	0.602
4 vs 1 ($S_1F_0Q_0$ vs $S_0F_0Q_0$)	1.08	[0.87,1.35]	0.470	0.529	1.05	[0.82,1.34]	0.713	0.847
5 vs 1 ($S_1F_1Q_1$ vs $S_0F_0Q_0$)	1.53	[1.24,1.89]	<0.001	<0.001	1.33	[1.05,1.67]	0.017	0.041

Notes: Model 1 controlled for all covariates, including age, gender, ethnicity, education, social class, wealth; Model 2 additionally controlled for the corresponding outcome measure at baseline (wave 4); p_{BH}, adjusted p value using the Benjamini and Hochberg (BH) procedure

© 2024 Bu F et al. *JAMA Network Open.*

eTable 6. Results From Regression Analysis Using Cluster 2 as the Reference (N=6,983, MI=30)

	Model 1				Model 2			
**Depression**	**OR**	**95% CI**	**p**	**p_{BH}**	**OR**	**95% CI**	**p**	**p_{BH}**
1 vs 2 ($S_0F_0Q_0$ vs $S_gF_0Q_1$)	1.23	[1.01,1.50]	0.036	0.071	1.26	[1.01,1.57]	0.041	0.110
3 vs 2 ($S_gF_1Q_1$ vs $S_gF_0Q_1$)	0.61	[0.49,0.75]	<0.001	0.000	0.83	[0.65,1.05]	0.121	0.229
4 vs 2 ($S_1F_0Q_0$ vs $S_gF_0Q_1$)	0.99	[0.80,1.21]	0.894	0.894	1.04	[0.83,1.32]	0.712	0.751
5 vs 2 ($S_1F_1Q_1$ vs $S_gF_0Q_1$)	0.45	[0.37,0.55]	<0.001	0.000	0.64	[0.52,0.80]	<0.001	<0.001
**Life Satisfaction**	**Coef.**	**95% CI**	**p**	**p_{BH}**	**Coef.**	**95% CI**	**p**	**p_{BH}**
1 vs 2 ($S_0F_0Q_0$ vs $S_gF_0Q_1$)	-1.57	[-2.14,-1.00]	<0.001	<0.001	-0.62	[-1.07,-0.17]	0.007	0.021
3 vs 2 ($S_gF_1Q_1$ vs $S_gF_0Q_1$)	1.58	[1.01,2.14]	<0.001	<0.001	-0.19	[-0.63,0.25]	0.389	0.492
4 vs 2 ($S_1F_0Q_0$ vs $S_gF_0Q_1$)	0.00	[-0.56,0.57]	0.996	0.996	-0.42	[-0.86,0.02]	0.062	0.097
5 vs 2 ($S_1F_1Q_1$ vs $S_gF_0Q_1$)	3.55	[3.03,4.07]	<0.001	<0.001	0.34	[-0.07,0.75]	0.100	0.135
**Quality of life: pleasure**	**Coef.**	**95% CI**	**p**	**p_{BH}**	**Coef.**	**95% CI**	**p**	**p_{BH}**
1 vs 2 ($S_0F_0Q_0$ vs $S_gF_0Q_1$)	-0.87	[-1.09,-0.66]	<0.001	<0.001	-0.26	[-0.44,-0.08]	0.004	0.015
3 vs 2 ($S_gF_1Q_1$ vs $S_gF_0Q_1$)	0.16	[-0.05,0.37]	0.128	0.164	-0.02	[-0.19,0.15]	0.812	0.858
4 vs 2 ($S_1F_0Q_0$ vs $S_gF_0Q_1$)	-0.29	[-0.50,-0.09]	0.005	0.010	-0.08	[-0.24,0.08]	0.339	0.617
5 vs 2 ($S_1F_1Q_1$ vs $S_gF_0Q_1$)	1.04	[0.85,1.22]	<0.001	<0.001	0.27	[0.11,0.42]	0.001	0.004
**Quality of life: self-realisation**	**Coef.**	**95% CI**	**p**	**p_{BH}**	**Coef.**	**95% CI**	**p**	**p_{BH}**
1 vs 2 ($S_0F_0Q_0$ vs $S_gF_0Q_1$)	-0.72	[-1.00,-0.44]	<0.001	<0.001	-0.15	[-0.38,0.07]	0.171	0.296
3 vs 2 ($S_gF_1Q_1$ vs $S_gF_0Q_1$)	0.49	[0.23,0.76]	<0.001	<0.001	0.11	[-0.09,0.32]	0.278	0.358
4 vs 2 ($S_1F_0Q_0$ vs $S_gF_0Q_1$)	-0.25	[-0.51,0.02]	0.067	0.086	-0.11	[-0.31,0.09]	0.283	0.358
5 vs 2 ($S_1F_1Q_1$ vs $S_gF_0Q_1$)	1.41	[1.16,1.65]	<0.001	<0.001	0.29	[0.10,0.48]	0.003	0.009
**Self-reported health**	**OR**	**95% CI**	**p**	**p_{BH}**	**OR**	**95% CI**	**p**	**p_{BH}**
1 vs 2 ($S_0F_0Q_0$ vs $S_gF_0Q_1$)	0.81	[0.66,1.00]	0.045	0.058	0.92	[0.72,1.19]	0.540	0.603
3 vs 2 ($S_gF_1Q_1$ vs $S_gF_0Q_1$)	0.95	[0.78,1.17]	0.636	0.646	0.85	[0.66,1.08]	0.179	0.312
4 vs 2 ($S_1F_0Q_0$ vs $S_gF_0Q_1$)	0.82	[0.67,1.00]	0.054	0.065	0.91	[0.71,1.17]	0.460	0.573
5 vs 2 ($S_1F_1Q_1$ vs $S_gF_0Q_1$)	1.43	[1.18,1.73]	<0.001	0.001	1.29	[1.03,1.62]	0.028	0.065
**Physical activity**	**OR**	**95% CI**	**p**	**p_{BH}**	**OR**	**95% CI**	**p**	**p_{BH}**
1 vs 2 ($S_0F_0Q_0$ vs $S_gF_0Q_1$)	0.94	[0.76,1.16]	0.547	0.616	1.00	[0.79,1.27]	0.996	0.996
3 vs 2 ($S_gF_1Q_1$ vs $S_gF_0Q_1$)	1.13	[0.91,1.40]	0.262	0.314	1.10	[0.87,1.40]	0.432	0.586
4 vs 2 ($S_1F_0Q_0$ vs $S_gF_0Q_1$)	1.02	[0.83,1.25]	0.878	0.901	1.05	[0.83,1.32]	0.691	0.847
5 vs 2 ($S_1F_1Q_1$ vs $S_gF_0Q_1$)	1.43	[1.18,1.75]	<0.001	0.001	1.33	[1.06,1.66]	0.013	0.030

Notes: Model 1 controlled for all covariates, including age, gender, ethnicity, education, social class, wealth; Model 2 additionally controlled for the corresponding outcome measure at baseline (wave 4); p_{BH}, adjusted p value using the Benjamini and Hochberg (BH) procedure

© 2024 Bu F et al. *JAMA Network Open.*

eFigure 1. Sample Selection Diagram

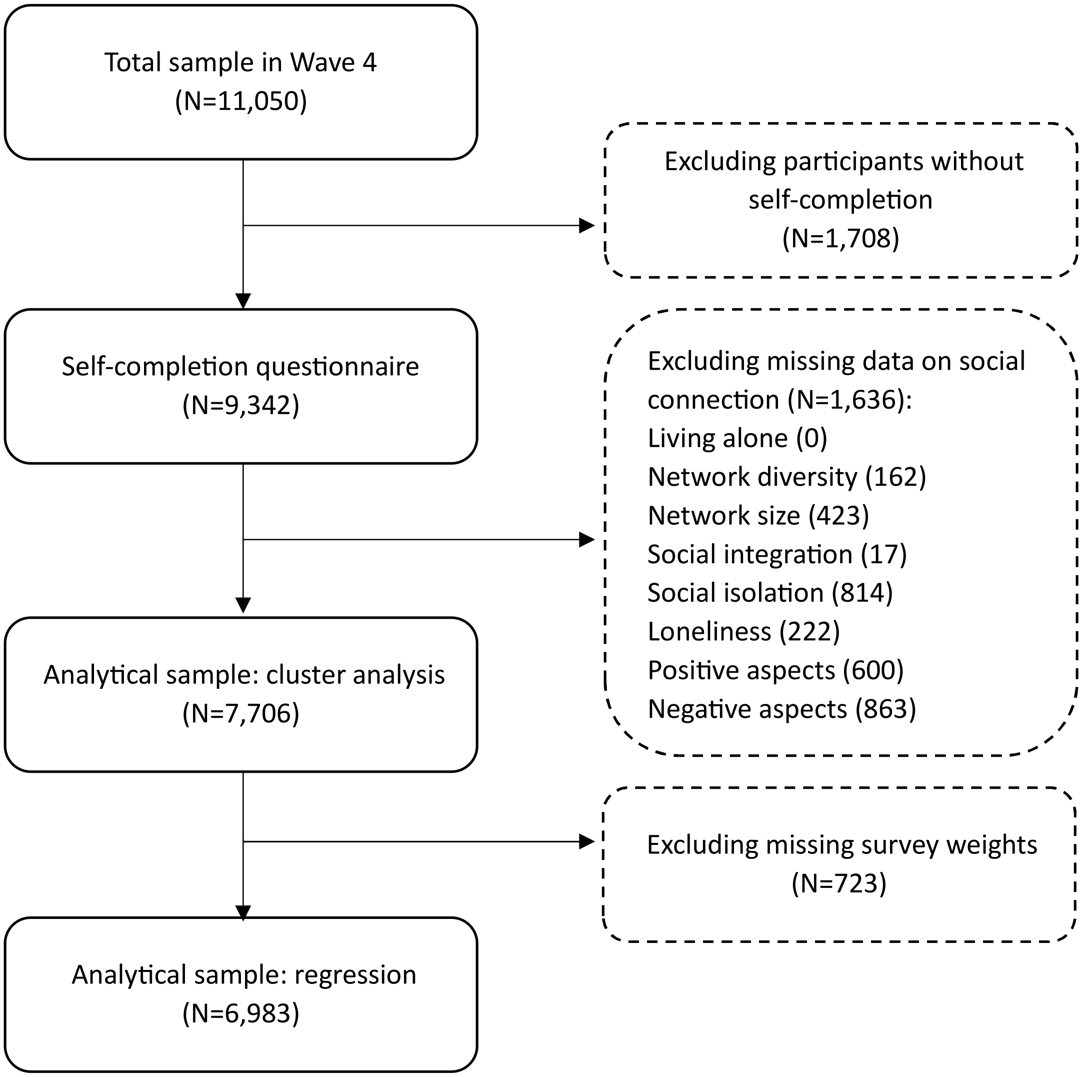

© 2024 Bu F et al. *JAMA Network Open.*

eFigure 2. Total Within-Clusters Sum of Squares by the Number of Clusters Using the K-Prototypes Algorithm and Integrated Completed Likelihood (ICL) Criterion Using Gaussian Mixture Model

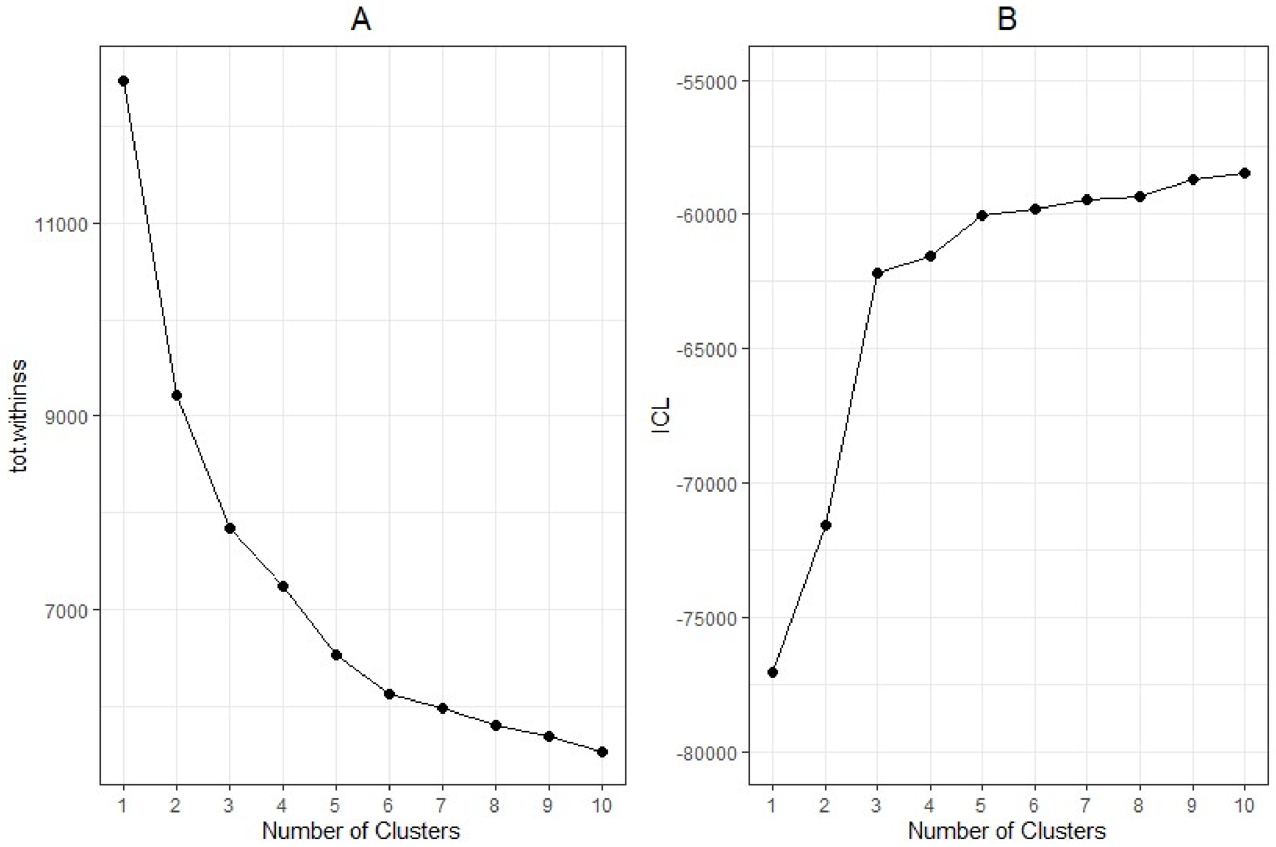

© 2024 Bu F et al. *JAMA Network Open.*

eFigure 3. Cluster Profiles Using the K-Prototypes Algorithm

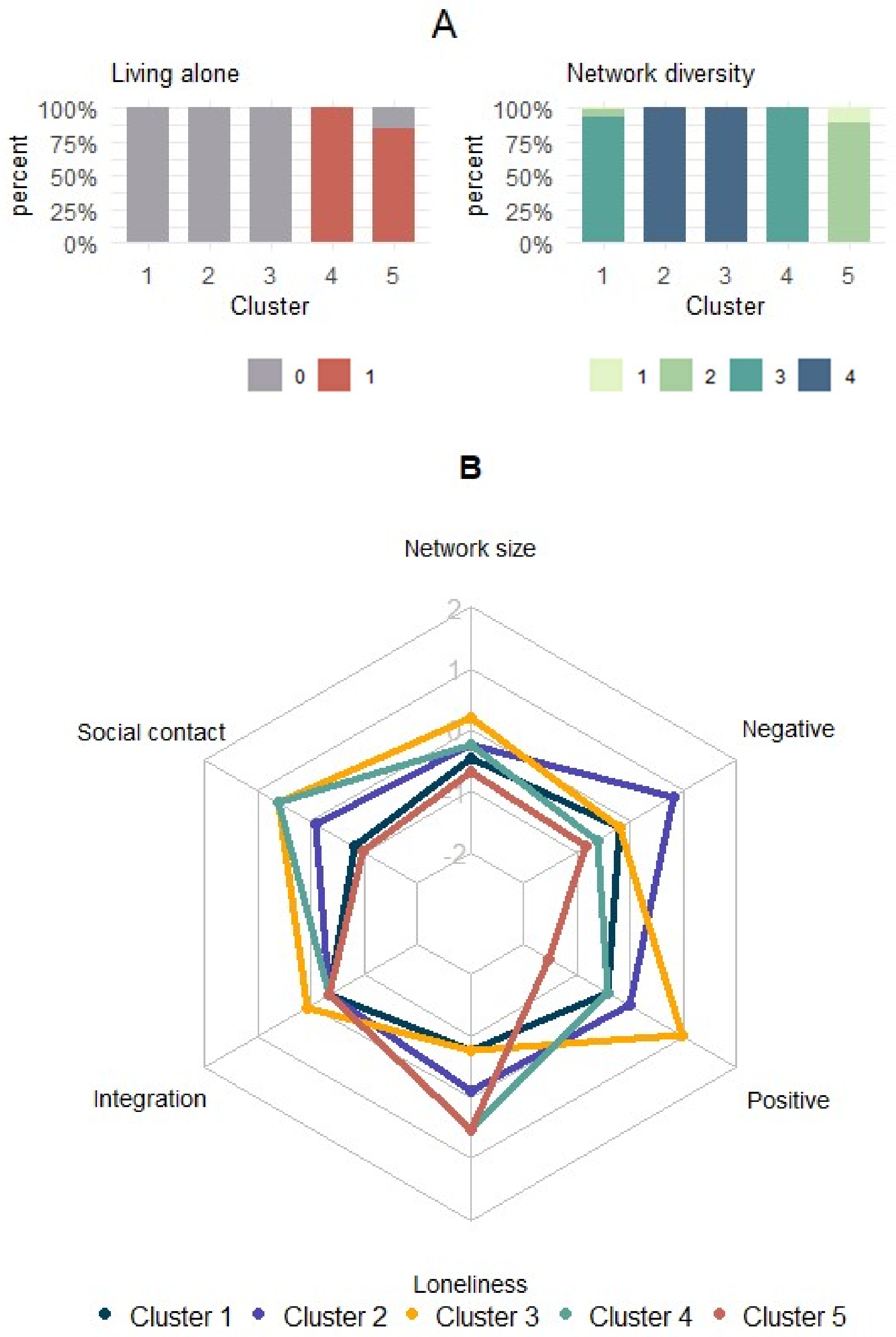

© 2024 Bu F et al. *JAMA Network Open.*

eFigure 4. Cluster Profiles Using Latent Class Model

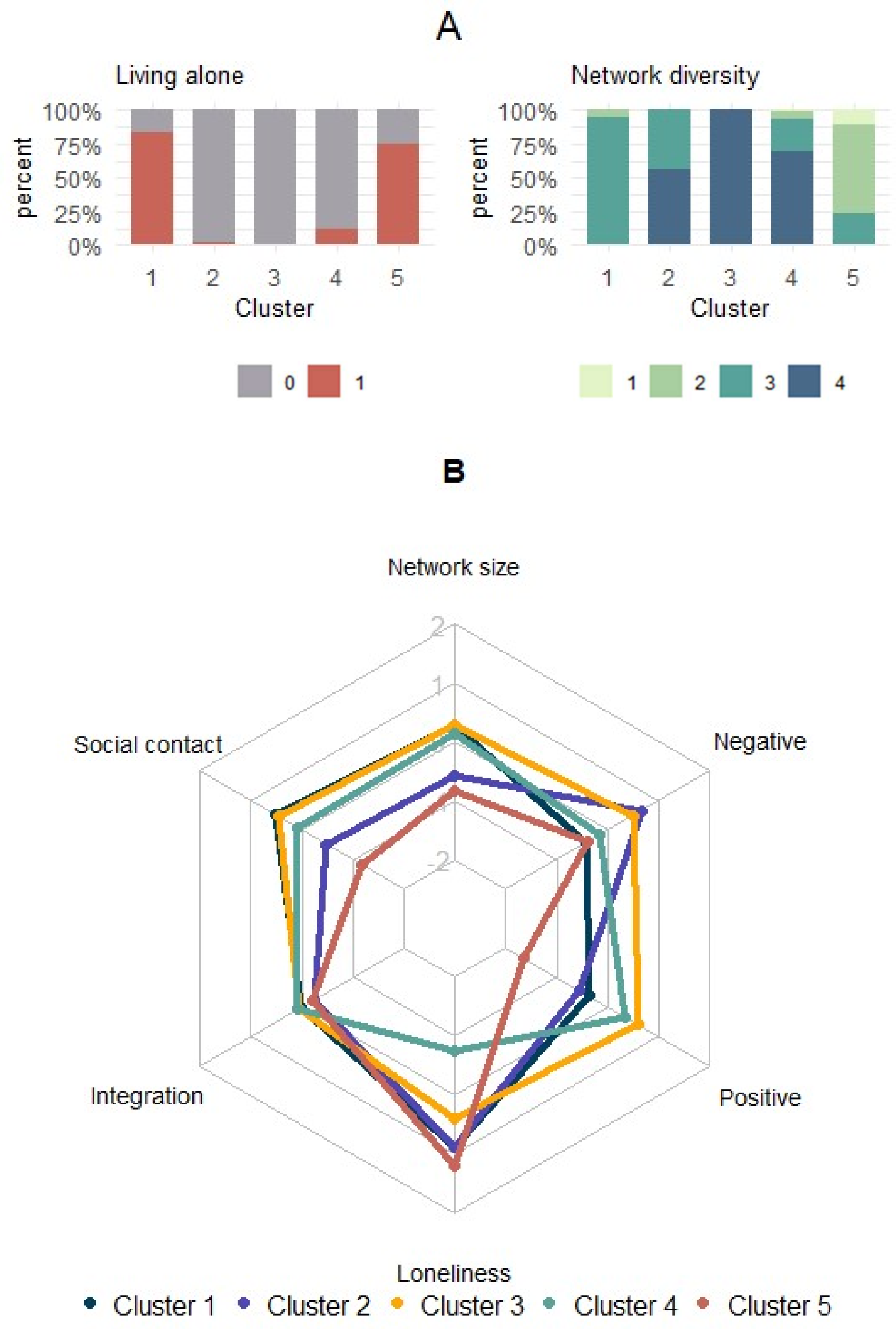

eFigure 4. Cluster Profiles Using Latent Class Model

© 2024 Bu F et al. *JAMA Network Open.*

eFigure 5. Linear Regression Residual Diagnostics

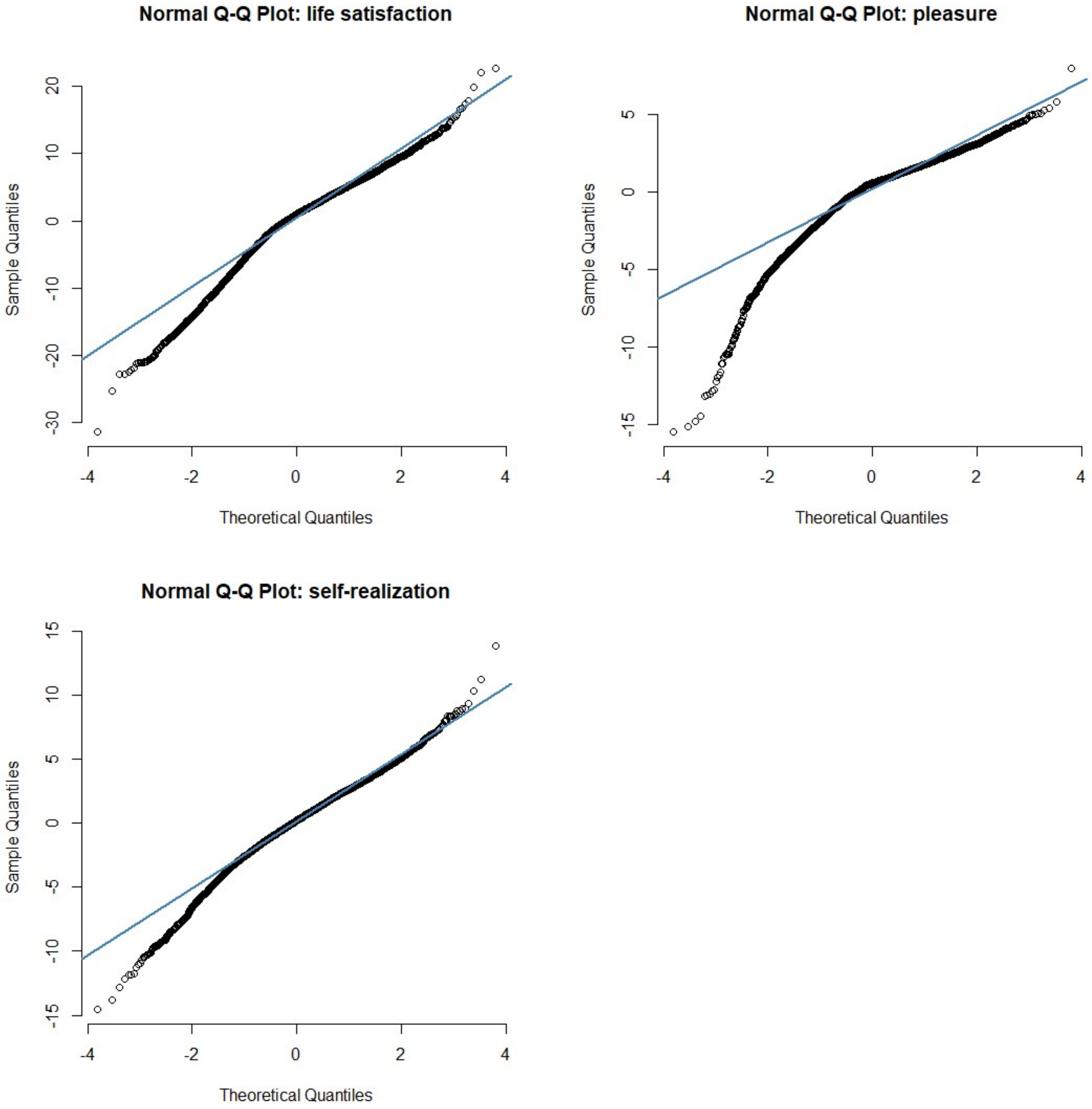

© 2024 Bu F et al. *JAMA Network Open.*
