# Supplementary material for: Patterns of Social Connection Among Older Adults in England
Source: JAMA Netw Open. 2024 Dec 23;7(12):e2451580. doi: 10.1001/jamanetworkopen.2024.51580 (PMC11667348; doi:10.1001/jamanetworkopen.2024.51580)
Supplement: Supplement 2. — Data Sharing Statement [file jamanetwopen-e2451580-s002.pdf]

Data Sharing Statement

Bu. Patterns of Social Connection Among Older Adults in England. *JAMA Netw Open*. Published December 23, 2024. doi:10.1001/jamanetworkopen.2024.51580

Data
Data available: Yes
Data types: Deidentified participant data
How to access data: The English Longitudinal Study of Ageing can be accessed via the UK Data Service: 10.5255/UKDA-SN-5050-27
When available: With publication

Supporting Documents
Document types: Statistical/analytic code
How to access documents: https://github.com/FFBU/ELSA_Cluster
When available: With publication

Additional Information
Who can access the data: Any researchers whose proposed use of the data has been approved.
Types of analyses: For research purpose
Mechanisms of data availability: Third party data archive
